# A new species of the genus *Boulenophrys* (Anura, Megophryidae) from Southwest China

**DOI:** 10.3897/BDJ.13.e153987

**Published:** 2025-07-03

**Authors:** Jing Liu, Shize Li, Yanlin Cheng, Gang Wei, Bin Wang, Gang Cheng

**Affiliations:** 1 Department of Resources and Environment, Moutai Institute, Renhuai, China Department of Resources and Environment, Moutai Institute Renhuai China; 2 Biodiversity Conservation Key Laboratory, Guiyang College, Guiyang, China Biodiversity Conservation Key Laboratory, Guiyang College Guiyang China; 3 Chengdu Institute of Biology, the Chinese Academy of Sciences, Chengdu, China Chengdu Institute of Biology, the Chinese Academy of Sciences Chengdu China

**Keywords:** Taxonomy, new species, molecular phylogenetic analysis, morphology

## Abstract

**Background:**

The Asian horned toad subfamily Megophryinae (Bonaparte, 1850) currently comprises more than 140 species and is widely distributed in southern China, as well as in Tropical Asia from India and Bhutan to the Philippines. During amphibian surveys conducted at Mt.Daxue Nature Reserve on June 28-30, 2023, we collected specimens of within the genus *Boulenophrys*. Based on molecular phylogenetic analyses and morphological comparisons, we describe this taxon as a new species from southwestern China.

**New information:**

Molecular phylogenetic analyses based on mitochondrial DNA strongly support the new species as a sister species of *B.jiangi*. The uncorrected genetic distances between the 16S rRNA and COI genes between the new species and its closest congener were 9.3% and 8.1%, respectively. The new species could be distinguished from its congeners by a combination of the following characters: (1) adult males have a moderate body size (SVL 37.1–40.6 mm), differing from *B.jiangi* in having longer hindlimbs when adpressed anteriorly—the tibiotarsal articulation reaches the mid-level of the eye when extended (vs. only reaching the area between the tympanum and the eye in *B.jiangi*); (2) vomerine ridge present and vomerine teeth absent; (3) tongue not notched behind; (4) a small horn-like tubercle at the edge of each upper eyelid; (5) tympanum distinctly visible, rounded; (6) toes lacking lateral fringes and webbing; (7) Distinct relative finger lengths: II < I < V < III in the new species (vs. I < II < V < III in *B.jiangi*); (8) heels overlapping when thighs are positioned at right angles to the body; (9) tibiotarsal articulation reaching the level of the middle of the eye when leg is stretched forward; (10) an internal single subgular vocal sac in male; (11) dense nuptial spines on dorsal bases of fingers I and II in breeding adult males;(12) Eye diameter (ED) significantly smaller than that of *B.jiangi* (ED: 4.00±0.28 in the new species vs. 5.00±0.38 in *B.jiangi*, P < 0.05).

## Introduction

The Asian horned toad subfamily Megophryinae (Bonaparte, 1850) currently comprises more than140 species and is widely distributed in southern China, Tropical Asia from India and Bhutan to China and south to the Sundas and the Philippines. (Frost 2025). The taxonomic arrangements especially on generic assignments of the group have been controversial for a long time (Chen et al. 2017, Delorme et al. 2006, Dubois et al. 2021, Fei et al. 2016, Li and Wang 2008, Li et al. 2020, Liu et al. 2018, Lyu et al. 2021, Lyu et al. 2023, Mahony et al. 2017, Qi et al. 2021, Rao and Yang 1997, Tian and Hu 1983). Recently, Lyu et al. (2023) reviewed the literature comprehensively and conducted morphological and molecular phylogenetic analyses, proposing a revised generic classification for Megophryinae. This classification divides this subfamily into ten genera: *Brachytarsophrys* Tian & Hu, 1983; *Atympanophrys* Tian & Hu, 1983; *Grillitschia* Dubois, Ohler & Pyron, 2021; *Sarawakiphrys* Lyu & Wang, 2023; *Jingophrys* Lyu & Wang, 2023; *Xenophrys* Günther, 1864; *Megophrys* Kuhl & Van Hasselt, 1822; *Pelobatrachus* Beddard, 1907;*Ophryophryne* Boulenger, 1903, and *Boulenophrys* Fei, Ye & Jiang, 2016. Among these genera, *Boulenophrys* is one of the most widely distributed and speciose taxa, which is distributed in "most of China except for Tibet, extending south into Vietnam, Laos, Thailand, Myanmar and the Philippines, with less certainty to Bhutan and northeastern India" (Frost 2025, Lyu et al. 2023). Southwestern China remains a global hotspot for amphibian diversity, with new species of the genus *Boulenophrys* continuing to be discovered and described in this region in recent years (Frost, 2025). Since 2020 alone, eight new Boulenophrys species have been documented in southwestern China, including *B.jiangi*, *B.qianbeiensis*, and others.

During amphibian surveys conducted at Mt. Daxue Nature Reserve from June 28–30, 2023, we collected specimens of a species within the genus *Boulenophrys*. The sampling effort involved three days of fieldwork focusing on montane streams and rocky habitats at elevations of 1,000–1,800 m. The survey area is characterised by complex topography with dense evergreen forests and perennial watercourses, typical of southwestern China's mountain ecosystems. Weather conditions during the surveys were predominantly overcast with intermittent rain, resulting in high air humidity (85–95%) that facilitated amphibian activity. Based on molecular phylogenetic analyses and morphological comparisons, we describe this taxon as a new species from southwestern China.

## Materials and methods

**Sampl ing**

**Figure 1.**
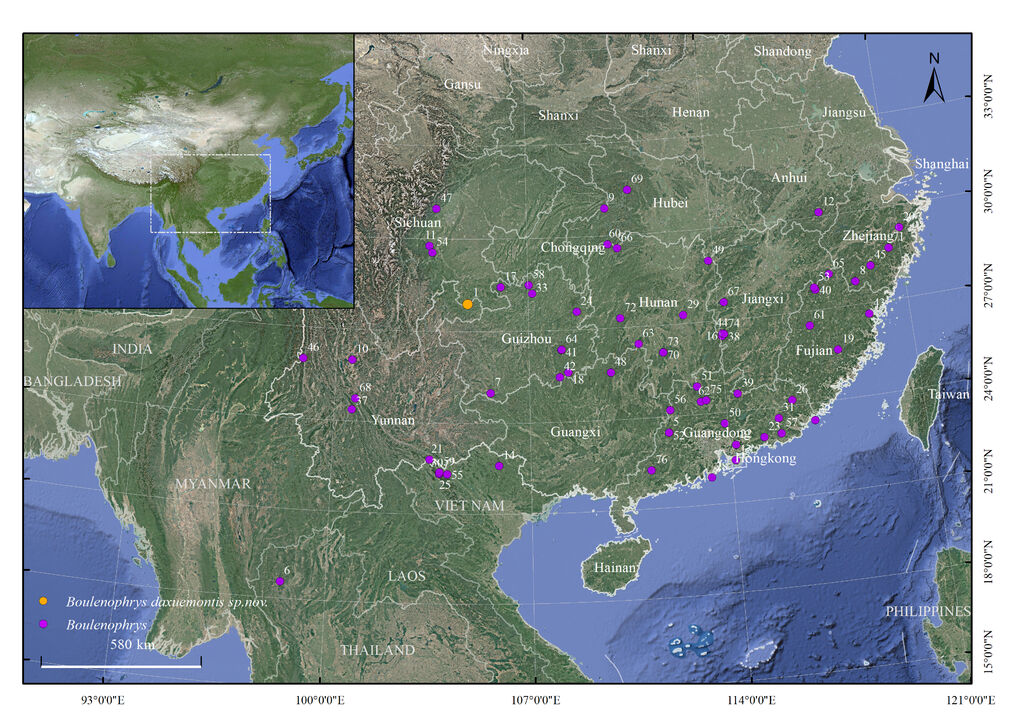
Map showing the distribution sites of *Boulenophrysdaxuemontis* sp. nov. The distribution information of the genus *Boulenophrys* is shown in Table 1, including newly recorded localities and previously published distributions. Base map downloaded from the China National Geomatics Center（https://www.tianditu.gov.cn/), satellite imagery and vector maps are from Google Maps(© 2025 Google, © TerraMetrics).China National Geomatics Center 2025,Google (2025)

In this study, five adult males of the undescribed species were collected at 9:00 PM on June 30, 2023 in a densely vegetated stream gully within Mt. Daxue Nature Reserve. (Fig. 1 and Table 1).The sampling site featured a gravel-bottomed streambed with loud flowing water, typical of montane forest habitats. Weather conditions at the time were overcast and rainy, with high air humidity (estimated 85–95%), which is conducive to amphibian activity at night. During the collection, multiple anuran calls were audible in the vicinity, with the most frequent and loudest vocalizations belonging to *Leptobrachellabijieensis*. The new species was found near the stream edge, either perched on moist rocks or hidden among leaf litter, consistent with the microhabitat preferences of congeners in *Boulenophrys*. In the field, the toads were euthanized by means of isoflurane(Herrel and Gibbon 2001), and the specimens were fixed in 10% buffered formalin and later transferred to 75% ethanol preservation. The muscle samples used for molecular analysis were preserved in 95% alcohol and stored at -20 ℃. The specimens collected in this work were deposited in Chengdu Institute of Biology, Chinese Academy of Sciences (CIB, CAS). All animal protocols in this study were approved by the Forestry Bureau of Junlian County, Sichuan Province (project number: JL5115202300054DC).The Animal Care and Use Committee of Guizhou University provided full approval for the research protocol (approval number: EAE-GZU-20244-T1228).

**Molecular data and phylogenetic analyses**

DNA was extracted from muscle tissue using a DNA extraction kit from Tiangen Biotech Co., Ltd. (Beijing). Two fragments of the mitochondrial 16S rRNA (16S) and cytochromeoxidase subunit I (COI) genes were amplifed. For 16S, the primers P7 (5’-CGCCTGTTTACCAAAAACAT-3’) and P8 (5’-CCGGTCTGAACTCAGATCACGT-3’) were used following Simon et al. (1994), and for COI, Chmf4 (5’-TYTCWACWAAYCAYAAAGAYATCGG-3’) and Chmr4 (5’-ACYTCRGGRTGRCCRAARAATCA-3’) were used following Che et al. (2012). Gene fragments were amplified under the following conditions: an initial denaturing step at 95 °C for 4 min; 36 cycles of denaturing at 95 °C for 30 s, annealing at 52 °C (for 16S)/47 °C (for COI) for 40 s and extending at 72 °C for 70 s. PCR products were purified with spin columns and then were sequenced with both forward and reverse primers, same as for the PCR. Sequencing was conducted using an ABI Prism 3730 automated DNA sequencer in Chengdu TSINGKE Biological Technology Co. Ltd. (Chengdu, China). All sequences were deposited in GenBank (see Table 1 for GenBank accession numbers).

For phylogenetic analyses, corresponding sequences of one *Xenophrysglandulosa* (Fei et al. 1990) and one *Xenophrysmangshanensis* (Fei et al. 1990) were also downloaded (Table 1), and used as outgroups following to Lin et al. (2024). Sequences were assembled and aligned using the Clustalw module in BioEdit v.7.0.9.0 (Hall 1999) with default settings. Alignments were checked by eye and revised manually if necessary. Trimming, with gaps partially deleted, was performed using GBLOCKS 0.91b (Castresana 2000). For phylogenetic analyses of mitochondrial DNA, the dataset was concatenated with 16S and COI gene sequences. Based on the 16S + COI concatenated dataset, phylogenetic analyses were conducted using maximum likelihood (ML) and Bayesian inference (BI) methods, implemented in PhyML 3.0 (Guindon et al. 2010) and MrBayes 3.12 (Ronquist and Huelsenbeck 2003), respectively, and the best-fit model was obtained by the Bayesian inference criteria (BIC) computed with PartitionFinder 2 (Lanfear et al. 2016). As a result, the analysis suggested that the best partition scheme is16S gene/each codon position of COI gene, and selected GTR + G + I model as the best model for each partition.

For ML analyses conducted in PhyML 3.0, the bootstrap consensus tree inferred from 1000 replicates was used to estimate nodal supports of inferred relationships on phylogenetic trees. For Bayesian analyses conducted in MrBayes 3.12, four Markov chains were run for 50 million generations with sampling every 1000 generations until the trees reached convergence (split frequency < 0.05). The first 25% of trees were removed as the “burn-in” stage followed by calculation of Bayesian posterior probabilities and the 50% majority-rule consensus of the post burn-in trees sampled at stationarity. Finally, mean genetic distance between *Boulenophrys* species based on uncorrected *p*-distance model was estimated on the16S and COI genes using MEGA v. 6.06 (Tamura et al. 2013).

**Morphological comparisons**

A total of thirteen specimens including five males of the new taxon and eight males of *B.jiangi* were measured (for voucher information see (Table 1). Additional morphological data was sourced from the literature (Table 2). The terminology and methods followed Fei et al. (2009). All specimens were measured by Jing Liu，measurements were taken with a dial caliper to 0.1 mm. Eighteen morphometric characters of adult specimens were measured(Table 3):

- **ED** eye diameter (distance from the anterior corner to the posterior corner of the eye);
- **FL** foot length (distance from tarsus to the tip of fourth toe);
- **HAL** hand length (distance from tip of third digit to proximal edge of inner palmar tubercle);
- **HDL** head length (distance from the tip of the snout to the articulation of jaw);
- **HDW** maximum head width (greatest width between the left and right articulations of jaw);
- **HLL** hindlimb length (maximum length from the vent to the distal tip of the Toe IV);
- **IND** internasal distance (minimum distance between the inner margins of the external nares);
- **IOD** interorbital distance (minimum distance between the inner edges of the upper eyelids);
- **LAL** length of lower arm and hand (distance from the elbow to the distal end of the Finger IV);
- **LW** lower arm width (maximum width of the lower arm);
- **SVL** snout-vent length (distance from the tip of the snout to the posterior edge of the vent);
- **SL** snout length (distance from the tip of the snout to the anterior corner of the eye);
- **TFL** length of foot and tarsus (distance from the tibiotarsal articulation to the distal end of the Toe IV);
- **THL** thigh length (distance from vent to knee),
- **TL** tibia length (distance from knee to tarsus);
- **TW** maximal tibia width;
- **TYD** maximal tympanum diameter;
- **UEW** upper eyelid width (greatest width of the upper eyelid margins measured perpendicular to the anterior-posterior axis).

In order to reduce the impact of allometry, the correct value from the ratio of each character to SVL was calculated and was log-transformed fo subsequent morphometric analysesLleonart et al. 2000. One-way analysis of variance (ANOVA) was used to test the significance of differences on morphometric characters between different species. To show the spatial distribution of different species on the morphometric characters, principal component analyses (PCA) were performed. The statistical analyses were performed using SPSS 21.0 (SPSS, Inc., Chicago. IL, USA), and differences were considered to be significant at p < 0.05. The undescribed species was also compared with all other *Boulenophrys* species on morphology (Table 4). Comparative data were obtained from related species as described in literature (Table 2).

## Data resources

All the sequences in this study were retrieved from GenBank and the accession numbers of the newly-determined sequences in this study are shown in (Table 1).

## Taxon treatments

### Boulenophrys daxuemontis

Liu, Li, Cheng, Wei, Wang & Cheng sp. nov.

919D2C09-1CC9-5050-936A-0212049E81E2

37558C8D-8AC9-4ADA-9512-F07CF9F0E9FB

The specific name *daxuemontis* refers to the distribution of this species, Daxue Mountain, Sichuan province, China.

#### Materials

- **Type status:** Holotype. **Occurrence:** recordedBy: Shi-Ze Li; sex: male; lifeStage: adult; occurrenceID: B17C4CFE-F2A9-5602-B987-2E04FCCD6FF8; **Taxon:** scientificName: *Boulenophrysdaxuemontis*; kingdom: Animalia; phylum: Chordata; class: Amphibia; order: Anura; family: Megophryidae; genus: Boulenophrys; **Location:** country: China; stateProvince: Sichuan; county: Junlian; verbatimElevation: 1455 m; decimalLatitude: 27.8952; decimalLongitude: 104.7665; **Event:** eventDate: 2023-06-30; **Record Level:** institutionID: CIB JL20230630024; collectionID: JL20230630024
- **Type status:** Paratype. **Occurrence:** recordedBy: Jing Liu; sex: male; lifeStage: adult; occurrenceID: 1B786FBC-55CE-509D-AE49-A57438C826FE; **Taxon:** scientificName: *Boulenophrysdaxuemontis*; kingdom: Animalia; phylum: Chordata; class: Amphibia; order: Anura; family: Megophryidae; genus: Boulenophrys; **Location:** country: China; countryCode: Sichuan; county: Junlian; verbatimElevation: 1455 m; decimalLatitude: 27.8952; decimalLongitude: 104.7665; **Identification:** identifiedBy: Shi-ze Li; **Event:** eventDate: 2023-6-30; **Record Level:** institutionID: CIB JL20230630025, CIB JL20230630026; collectionID: JL20230630025, JL20230630026
- **Type status:** Paratype. **Occurrence:** recordedBy: Jing Liu; occurrenceID: 64D3CC95-DC9F-5164-AEE4-FEFA569996EC; **Taxon:** scientificName: *Boulenophrysdaxuemontis*; kingdom: Animalia; phylum: Chordata; class: Amphibia; order: Anura; family: Megophryidae; genus: Boulenophrys; **Location:** country: China; stateProvince: Yunnan; county: Weixin; verbatimElevation: 1495 m; decimalLatitude: 27.8879; decimalLongitude: 104.7695; **Identification:** identifiedBy: Shi-ze Li; **Event:** eventDate: 2023-06-30; **Record Level:** institutionID: CIB WX20230630006, CIB WX20230630007; collectionID: WX20230630006, WX20230630007

#### Description

**Figure 2.**
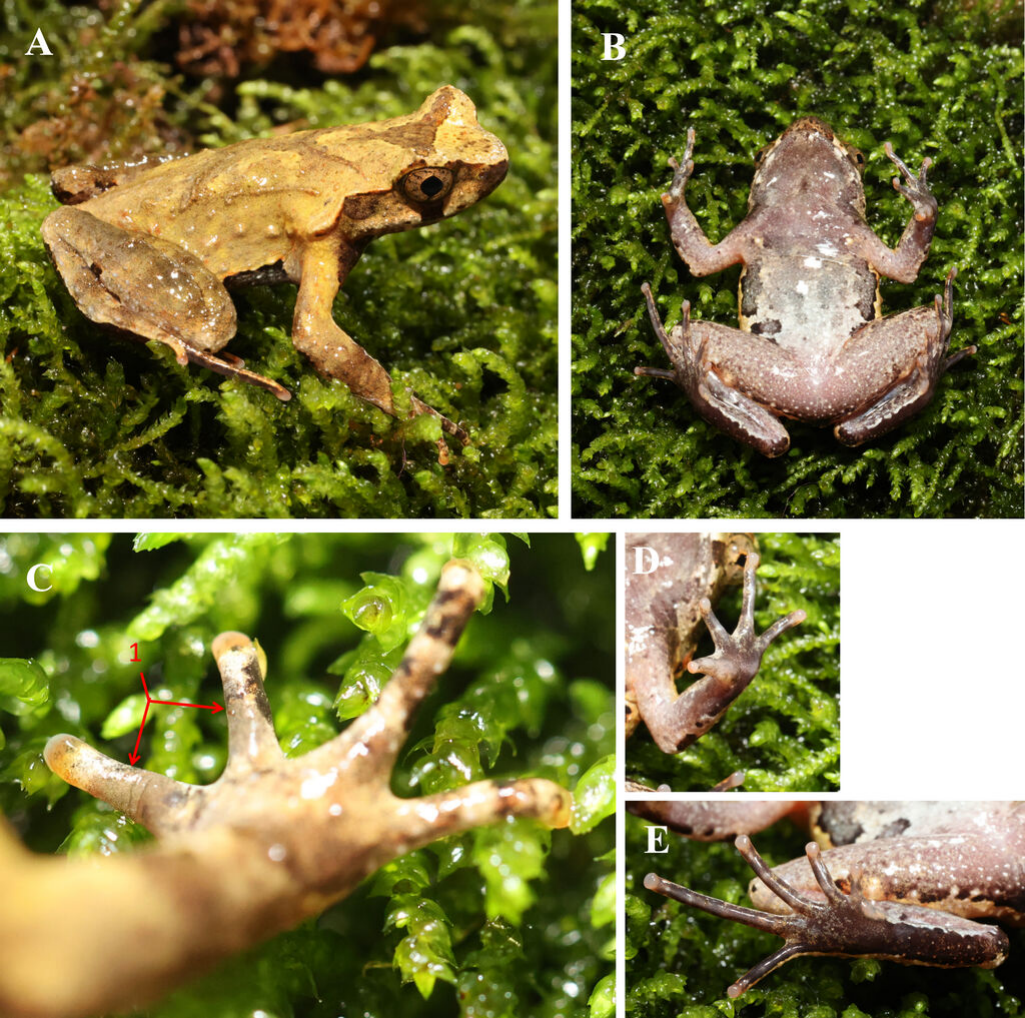
Photos of the adult male holotype CIB JL20230630024 of *Boulenophrysdaxuemontis* sp. nov. in life. **A** dorsal view; **B** ventral view; **C** dorsal view of hand showing nuptial pads on the first and second fingers (1); **D** ventral view of hand; **E** ventral view of foot.

**Figure 3.**
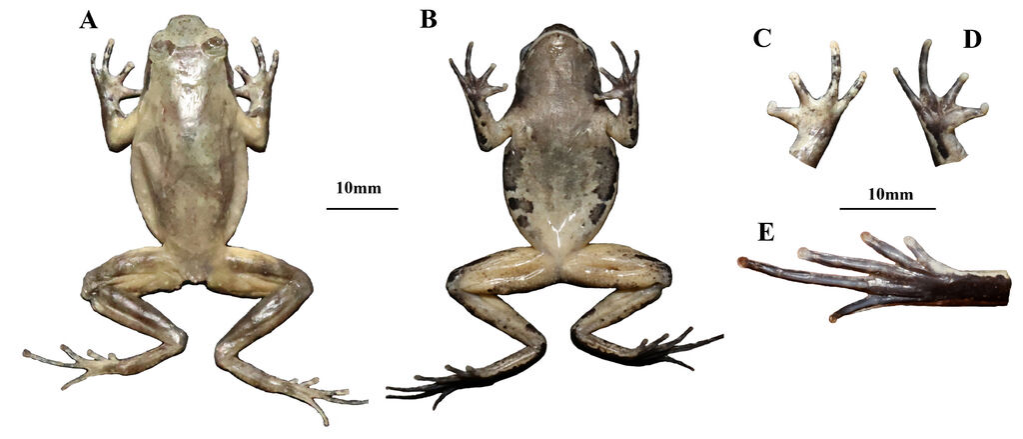
The holotype specimen CIB JL20230630024 of *Boulenophrysdaxuemontis* sp. nov. in preservation. **A** dorsal view; **B** ventral view; **C** dorsal view of right hand; **D** ventral view of right hand; **E** ventral view of right foot.

**Description of holotype** (Fig. 2, Fig. 3). SVL 37.7 mm; head width larger than head length (HDW/HDL ratio about 1.1); snout obtusely pointed, protruding well beyond the margin of the lower jaw in ventral view; loreal region vertical and concave; canthus rostralis well-developed; top of head flat in dorsal view; eye large, eye diameter 38.7% of head length; pupils vertical; nostril rounded, distinct, closer to snout than eye; tympanum distinct, TYP/EYE ratio 0.49; Vomerine ridges are distinctly V-shaped, and vomerine teeth are absent; have maxillary teeth; margin of tongue smooth, not notched behind; adult males have a single subgular vocal sac.

Forelimbs slender, the length of lower arm and hand 42.1% of SVL; fingers burly, relative finger lengths: II < I < V < III; tips of digits globular, without lateral fringes; subarticular tubercle distinct at the base of each finger; two metacarpal tubercles, oval-shaped, the inner one bigger than the outer one.

Hindlimbs slender, 1.4 times of SVL; heels overlapping when thighs are positioned at right angles to the body, tibiotarsal articulation reaching the level of middle eye when leg stretched forward; tibia length longer than thigh length; relative toe lengths I < II < V < III < IV; tips of toes round, slightly dilated; subarticular tubercles present on the base of each toe; toes without interdigital webbing and lateral fringe; inner metatarsal tubercle long ovoid and outer metatarsal tubercle absent; dense nuptial spines on dorsal bases of fingers I and II in breeding adult males.

Dorsal skin rough, numerous granules scattered on the surface of the head, the dorsum and the limbs; several large warts scattered on flanks; tubercles on the dorsum forming a X-shaped ridge; two discontinuous dorsolateral parallel ridges on either side of the X-shaped ridges; an inverted triangular brown speckle between two upper eyelids; single horn-like prominent tubercle on edge of upper eyelid; supratympanic fold distinct; temporal region excluding tympanum, upper eyelid and surface around cloaca with conical tubercles; surface of throat, chest and abdomen smooth; dense rounded tubercles on ventral thighs; small pectoral gland closer to axilla and bigger femoral gland positioned on posterior surface of thigh at midpoint between knee and cloaca.

**Colouration of holotype in life.** (Fig. 2). Dorsal surfaces of head and trunk brownish-yellow; an inverted triangular brown speckle between the eyes; X-shaped ridges on central dorsum; supratympanic fold light brown; three light brown transverse bands on dorsal surfaces of thigh and shank; 4 dark brown and white vertical bars on lower and upper lip; dark vertical band below eye; throat, anterior chest and surface of limbs light purple-brown; anterior belly light grey and posterior white and large black patches on belly sides, forming a discontinuous line; some white granules on the ventral surfaces of hindlimbs; dorsal surfaces of hind limbs and toes light brown with orange spots and dark brown transverse bands; palms and ventral surfaces of toes black-brown and the tip of fingers grey-white.

**Colouration of holotype in preservation.** (Fig. 3). After preservation in ethanol, dorsal surfaces light brownish grey; X-shaped ridges on dorsum indistinct and inverted triangular brown speckle between the eyes, transverse bands on limbs and digits distinct; surface of throat and anterior chest dark black-brown; anterior belly light grey-white and posterior belly grey-white; inner thigh, and upper part of tibia milky yellow; palms, ventral surfaces of soles and toes dark brown; inner metatarsal tubercle milky yellow.

**Figure 4.**
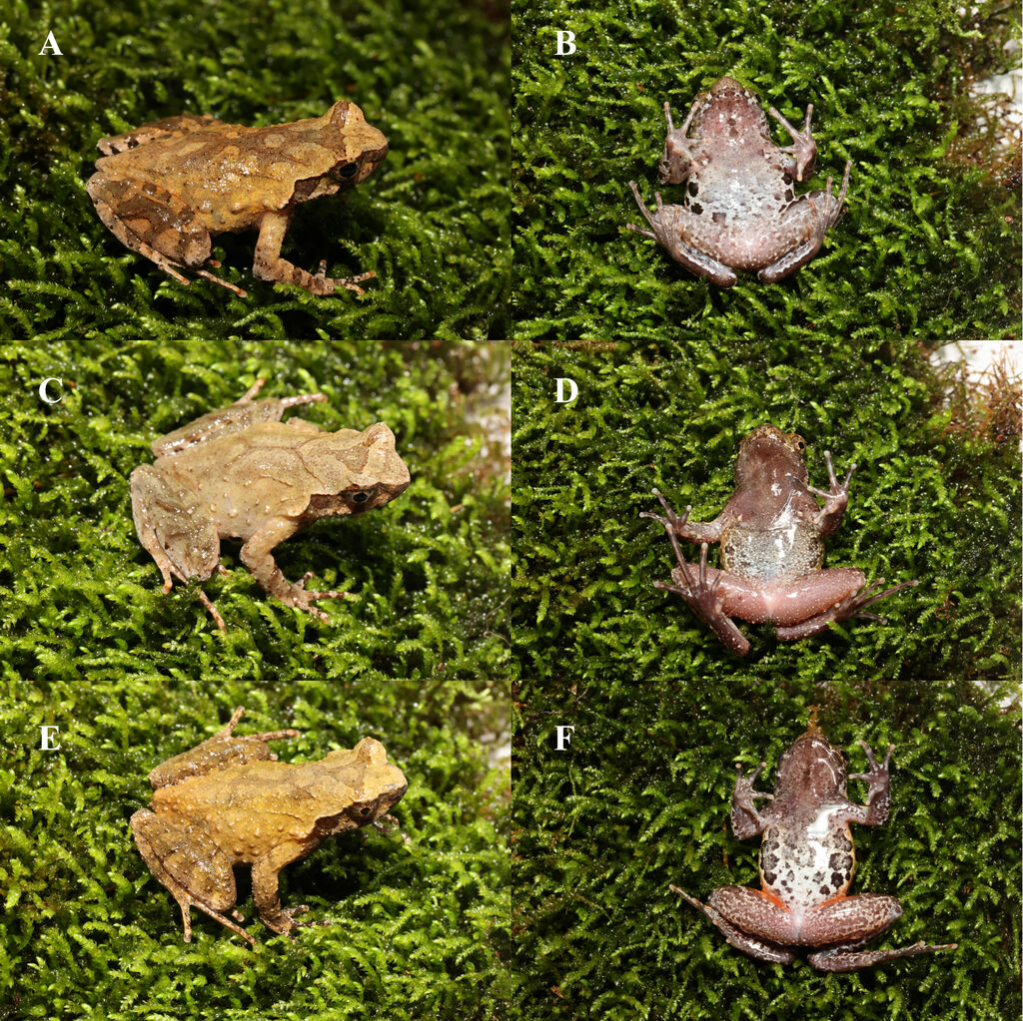
Color variation in *Boulenophrysdaxuemontis* sp. nov. Dorsal and ventral views of male specimen CIB WX20230630007 (**A, B**); dorsal and ventral views of male specimen CIB JL20230630025 (**C, D**); dorsal and ventral views of male specimen CIB JL20230630026 (**E, F**).

**Variation.** (Fig. 4). In specimen CIB WX20230630007 the dorsolateral parallel ridges are short, the dark - brown colour patches are darker, and the abdominal spots are lighter and whiter (Fig. 4A, B); in specimen CIB JL20230630025 the colour patches on the back are lighter, making the "X - shaped ridges" more prominent, the warts on the sides are denser (Fig. 4C), the black spots on the abdomen are smaller, and the white spots on the inner side of the legs are sparser (Fig. 4D); in specimen CIB JL20230630026 the ridges on the back are shorter, and the warts on the sides are denser (Fig. 4E), the black patches on the abdomen are larger and cover the entire abdomen,the posterior ends of both sides of the abdomen and the outer sides of the thighs exhibit an orange-red colour. (Fig. 4F).

**Comparisons.** *Boulenophrysdaxuemontis* sp. nov. can be distinguished from *B.binlingensis*, *B.caudoprocta*, *B.fanjingmontis*, *B.jingdongensis*, *B.mirabilis*, *B.omeimontis*, *B.qianbeiensis*, *B.sangzhiensis*, *B.shuichengensis*, *B.spinata* by having a medium body size in males (maximum SVL < 41.0 mm in males vs minimum SVL > 45.0 mm in all other species). And differs from *B.acuta*, *B.angka*, *B.cheni*, *B.daiyunensis*, *B.elongata*, *B.frigida*, *B.gaolanensis*, *B.hengshanensis*, *B.hungtai*, *B.kuatunensis*, *B.mufumontana*, *B.rubrimera*, *B.sanmingensis*, *B.shimentaina*, *B.tongboensis* and *B.wuliangshanensis* by having a medium body size in males (minimum SVL > 37.0 mm in males vs maximum SVL < 33.0 mm in all other species) (Suppl. material 1).

*Boulenophrysdaxuemontis* sp. nov. can be distinguished from *B.acuta*, *B.caudoprocta*, *B.jinggangensis*, *B.liboensis*, *B.mirabilis*, *B.palpebralespinosa* and *B.shuichengensis* by having a small horn-like tubercle at the edge of each upper eyelid vs. having a prominent and elongated tubercle in the latter. And differs from *B.binchuanensis*, *B.binlingensis*, *B.minor*, *B.spinata*, *B.wuliangshanensis* and *B.wushanensis* by having a small horn-like tubercle at the edge of each upper eyelid vs. absent in the latter.

*Boulenophrysdaxuemontis* sp. nov. can be distinguished from *B.acuta*, *B.anlongensis*, *B.baishanzuensis*, *B.binchuanensis*, *B.boettgeri*, *B.cheni*, *B.congjiangensis*, *B.daiyunensis*, *B.daoji*, *B.fanjingmontis*, *B.jingdongensis*, *B.jinggangensis*, *B.kuatunensis*, *B.liboensis*, *B.lini*, *B.lushuiensis*, *B.mirabilis*, *B.mufumontana*, *B.nanlingensis*, *B.omeimontis*, *B.palpebralespinosa*, *B.qianbeiensis*, *B.rubrimera*, *B.sangzhiensis*, *B.sanmingensis*, *B.shimentaina*, *B.shuichengensis*, *B.spinata*, *B.wushanensis*, *B.xiangnanensis* and *B.yangmingensis* by toes without lateral fringes vs. toes with lateral fringes in the latter.

*Boulenophrysdaxuemontis* sp. nov. can be distinguished from *B.acuta*, *B.angka*, *B.anlongensis*, *B.binchuanensis*, *B.binlingensis*, *B.boettgeri*, *B.brachykolos*, *B.caobangensis*, *B.caudoprocta*, *B.cheni*, *B.congjiangensis*, *B.daiyunensis*, *B.daoji*, *B.dongguanensis*, *B.dupanglingensis*, *B.fanjingmontis*, *B.fengshunensis*, *B.hengshanensis*, *B.insularis*, *B.jingdongensis*, *B.jinggangensis*, *B.jiulianensis*, *B.leishanensis*, *B.liboensis*, *B.lini*, *B.lushuiensis*, *B.minor*, *B.mirabilis*, *B.mufumontana*, *B.nankunensis*, *B.nanlingensis*, *B.obesa*, *B.omeimontis*, *B.palpebralespinosa*, *B.puningensis*, *B.qianbeiensis*, *B.sangzhiensis*, *B.sanmingensis*, *B.shimentaina*, *B.shuichengensis*, *B.shunhuangensis*, *B.spinata*, *B.tuberogranulata*, *B.wugongensis*, *B.wushanensis*, *B.xianjuensis*, *B.xiangnanensis*, *B.xuefengmontis*, *B.yangmingensis*, *B.yaoshanensis*, *B.yingdeensis* and *B.yunkaiensis* by toes without webbing vs. having rudimentary webbing or most one-fourth webbed.

*Boulenophrysdaxuemontis* sp. nov. is phylogenetically closest to *B.jiangi*，in the same branch as *B.chishuiensis* and *B.minor*. This new species could be distinguished from *B.jiangi* distinctly by tibiotarsal articulation reaching the level of middle eye (vs. between tympanum to eye), relative finger lengths: II < I < V < III (vs. I < II < V < III) ,vomerine ridges present (vs. absent) and having significantly higher ratios of IOD to SVL and lower ratios of ED, TL and FL to SVL (Table 4).This new species could be identified from *B.chishuiensis* distinctly by having smaller body size SVL 37.1-40.6mm in males (vs. SVL 43.4-44.1mm in males); tibiotarsal articulation reaching the level of middle eye (vs. between tympanum to eye). This new species could be identified from *B.minor* distinctly by relative finger lengths: II < I < V < III (vs. I = II < V < III) , tongue not notched behind (vs. notched in the latter), toes without webbing (vs.having rudimentary webbing).Suppl. material 1

**Secondary sexual characters.** Adult males have a single subgular vocal sac and dense nuptial spines on dorsal bases of fingers I and II in breeding adult males (Fig. 2C).

#### Diagnosis

*Boulenophrysdaxuemontis* sp. nov. is assigned to the genus *Boulenophrys* based on molecular phylogenetic analyses and the following generic diagnostic characters: snout shield-like; projecting beyond the lower jaw; canthus rostralis distinct; chest glands small and round, closer to the axilla than to midventral line; femoral glands on rear part of thigh; vertical pupils (Fei et al. 2016).

*Boulenophrysdaxuemontis* sp. nov. can be distinguished from its congeners by a combination of the following morphological characters: (1) body size moderate (SVL 37.1-40.6 mm in males); (2) vomerine ridge present and vomerine teeth absent; (3) tongue not notched behind; (4) a small horn-like tubercle at the edge of each upper eyelid; (5) tympanum distinctly visible, rounded; (6) toes without lateral fringes and webbing; (7) heels overlapped when thighs are positioned at right angles to the body; (8) tibiotarsal articulation reaching the level of middle eye when leg stretched forward; (9) an internal single subgular vocal sac in male; (10) dense nuptial spines on dorsal bases of fingers I and II in breeding adult males.

#### Etymology

The specific name *daxuemontis* refers to the distribution of this species, Daxue Mountain. We propose the common name “*daxueshan* horned toad” (English) and 大雪山角蟾 (Chinese).

#### Distribution

*Boulenophrysdaxuemontis* sp. nov. is known from the Daxue Mountain, which is situated at the border between Sichuan and Yunnan provinces in China; it was collected at elevations between 1400–1500 m. The species is distributed in the Daxueshan Nature Reserve, where the habitat features dense vegetation and high forest coverage. Due to its remote location, the intensity of human disturbance is relatively low. During several days of field surveys, *Boulenophrysdaxueshanensis* sp.nov. was found on both sides of Daxueshan Mountain, indicating that it occupies a specific distribution area within the reserve. No distinct vocalisations were heard at several distribution sites, and while other sympatric species have larger populations, no tadpoles or other life stages were found during the surveys, suggesting that the population size of this species is small. Therefore, population surveys and conservation efforts should be strengthened.

#### Ecology

**Figure 5.**
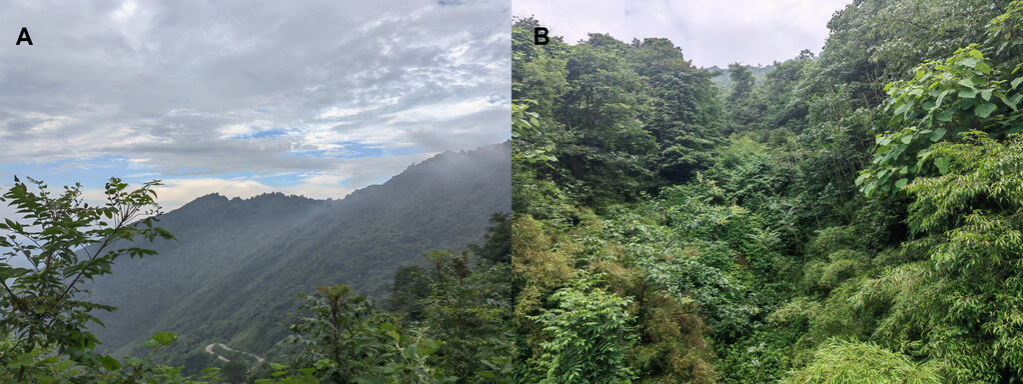
Habitats of *Boulenophrysdaxuemontis* sp. nov. in the type locality, Daxue Mountain, Junlian County, Sichuan Province, China. **A** landscape of montane forests in the type locality; **B** the new species inhabits a mountain stream that is completely covered by vegetation.

The individuals of the new species were frequently found on stones in the streams surrounded by evergreen broadleaved forests (Fig. 5), and tree sympatric amphibian species, i.e. *B.qianbeiensis* (Su et al. 2020), *Quasipaaboulengeri* (Günther 1889) and *Leptobrachellabijie* Wang, Li, Li, Chen, and Wang, 2019 were found. The survey was conducted from 8 to 10 p.m. on an overcast, rainy day, with air humidity ranging from 85% to 95% and a temperature of approximately 16°C. The habitat streams had a gravel substrate, narrow width, but significant elevation drop, resulting in fast-flowing water with distinct rushing sounds. While various frog calls were audible near the habitat—particularly the pronounced and intense vocalisations of *Leptobrachellabijie*, which overwhelmed other sounds—no valid vocalisations of the new species were collected. Extensive searches in multiple nearby streams and gullies failed to detect tadpoles of the new species.

## Analysis

**Phylogenetic analyses**

**Figure 6.**
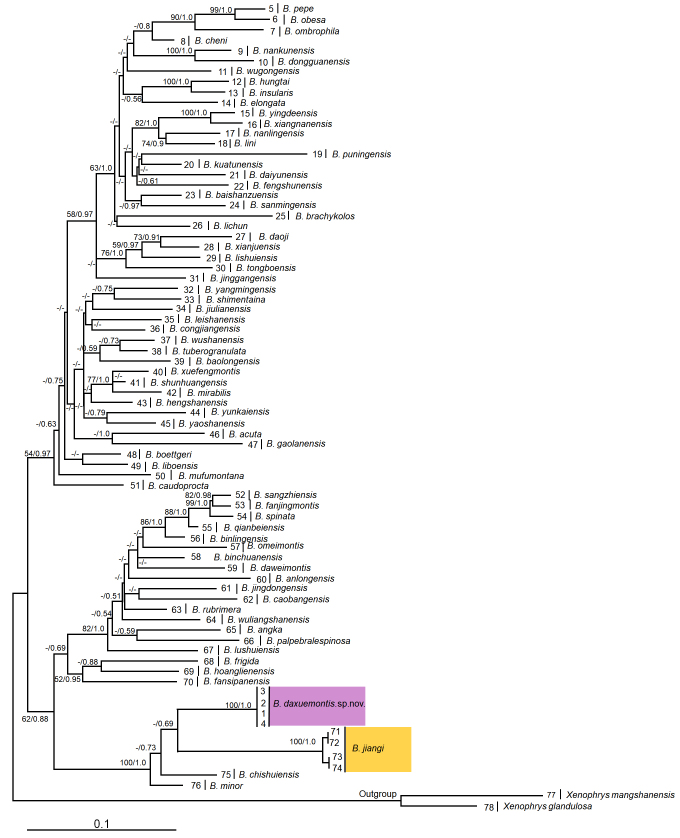
Maximum likelihood (ML) tree of the genus *Boulenophrys* reconstructed based on the 16S rRNA and COI gene sequences. Bayesian posterior probability/ML bootstrap supports were denoted beside each node. Samples 1–78 refer to Table 1.

Aligned sequence matrix of 16S+COI contains 1104 bp. ML and BI trees of the mitochondrial DNA dataset presented almost consistent topology (Fig. 6), and the undescribed species was clustered as a sister species with *B.jiangi* with low approval ratings.

The genetic distances of the 16S gene between samples of the undescribed species, based on the uncorrected p-distance model, range from 0.1% to 0.6%. The genetic distance between the undescribed species and its closest related species *B.yangmingensis* was 4.7% on 16S gene, which was higher or equal to those among many pairs of congeners, for example, 0.9% between *B.binlingensis* and *B.fanjingmontis*, and 0.9% between *B.fanjingmontis* and *B.qianbeienis* (Suppl. material 2). The genetic distances of the COI gene between samples of the undescribed species, based on the uncorrected p-distance model, range from 0% to 0.2%. The genetic distance between the undescribed species and its closest related species *B.minor* was 6.6% on COI gene, which was higher or equal to those among many pairs of congeners, for example, 4.1% between *B.spinata* and *B.fanjingmontis*, 3.1% between *B.fanjingmontis* and *B.sangzhiensis*, and 3.6% between *B.spinata* and *B.sangzhiensis* (Suppl. material 3).

**Morphological comparisons**

**Figure 7.**
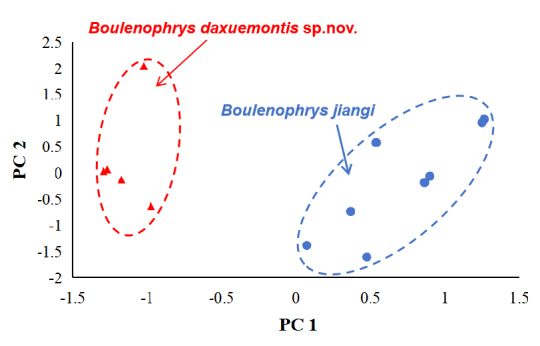
Plots of the first principal component (PC1) versus the second (PC2) for *Boulenophrysdaxuemontis* sp. nov. and *B.jiangi* from principal component analyses on male group.

The results of one-way ANOVA indicated that in males, the new taxon was significantly different from *B.jiangi* on many morphometric characters such as IOD, ED, TL and FL (all *p*-values < 0.05; Table 4). In PCA for males, we retained the first two principal components that accounted for 50.1% of the total variance (Table 5). Loadings for PC1, which accounted for 28.8% of the total variance, were most heavily loaded on IOD, THL and TL, and loadings for PC2, which accounted for 21.3%, were heavily loaded on maximal LAL. Differentiation was found along the PC1 axis between the newly collected specimens and *B.jiangi* (Fig. 7). More detailed descriptions of results from morphological comparisons between the new taxon and its congeners were presented in the following sections for describing the new species.

Based on the molecular and morphological diﬀerences, the specimens from Daxue Mountain, Sichuan and Yunan Province, China, represent a new species which is described as *Boulenophrysdaxuemontis* sp. nov.

## Discussion

Based on morphological characteristics and phylogenetic relationships, we identified a new species in the genus *Boulenophrys*. The genetic distance of the 16S rRNA gene between this new species and its sister species *B.jiangi* reaches 9.3%, which is significantly higher than the interspecific genetic distances of other congeneric species. Additionally, the COI genetic distance is 8.1%, which is smaller than that of 16S. This phenomenon contradicts the topological structure of the phylogenetic tree. It is hypothesized that this contradiction may be related to mountainous geographic isolation and asymmetric evolutionary driving mechanisms(Jin et al. 2022).

The Wumeng Mountains, where the study area is located, lie on the eastern side of the Hengduan Mountains. Their rugged canyon terrain forms a geographic barrier that not only isolates gene flow between animal populations but also promotes local population differentiation through genetic drift, ultimately creating evolutionary islands with significant genetic differences (Jiang et al. 2019). It is worth noting that the restricted distribution of species may lead to inflated genetic distances due to the accelerated differentiation of mitochondrial genes. Specifically, the long-branch effect in genetic distance calculations may amplify this phenomenon—although the rapidly evolving 16S gene shows large genetic differences, the slower-evolving COI gene can still effectively reflect the true sister-species relationships. This pattern is consistent with research results on Bufo species complexes (Fu et al. 2017).

Phylogenetic analyses show that the newly discovered Boulenophrysdaxueshanensis is topologically close to *B.jiangi and B.chishuiensis*, but significant morphological differences exist among the three, and their geographic distributions are effectively isolated by canyon barriers formed by water systems such as the Chishui River and Wujiang River. As an important biogeographic unit on the eastern side of the Hengduan Mountains, the Wumeng Mountain area demonstrates potential as a hotspot for cryptic species differentiation in amphibians(Zhu et al. 2022). In the future, genetic diversity assessments and niche analyses should be prioritised to provide a scientific basis for formulating conservation strategies.

## Supplementary Material

XML Treatment for Boulenophrys daxuemontis

7FE6CA27-20A4-5D09-B7D5-0C29FF70AA22 10.3897/BDJ.13.e153987.suppl1 Supplementary material 1 Diagnostic characters Data type morphological Brief description Diagnostic characters separating *Boulenophrysdaxuemontis* sp. nov. from other species of *Boulenophrys*. File: oo_1332637.xlsx https://binary.pensoft.net/file/1332637 Jing Liu, Shi-Ze Li, Yan-Lin Cheng, Gang Wei, Bin Wang, Gang Cheng

74A12216-9CE8-58B1-978C-24AD81FE64BA 10.3897/BDJ.13.e153987.suppl2 Supplementary material 2 Uncorrected *p*-distances based on 16S gene sequences Data type phylogenetic Brief description Uncorrected *p*-distances between the *Boulenophrys* species based on the 16S gene sequences. File: oo_1279899.xls https://binary.pensoft.net/file/1279899 Jing Liu, Shi-Ze Li, Yan-Lin Cheng, Gang Wei, Bin Wang, Gang Cheng

801874B6-B4AE-5A8A-A102-318BFF3180D4 10.3897/BDJ.13.e153987.suppl3 Supplementary material 3 Uncorrected *p*-distances based on COⅠ gene sequences Data type phylogenetic Brief description Uncorrected *p*-distances between the *Boulenophrys* species based on the COⅠ gene sequences. File: oo_1332638.xls https://binary.pensoft.net/file/1332638 Jing Liu , Shi-Ze Li, Yan-Lin Cheng, Gang Wei, Bin Wang, Gang Cheng

## Figures and Tables

**Table 1.** Table 1Information for samples used in molecular phylogenetic analyses in this study.

ID	*Boulenophrys* species	Voucher number	Locality	16S	COI
1	*Boulenophrysdaxuemontis* sp.nov.	CIB JL20230630024	China, Sichuan, Junlian County,Mt Daxue	PV469383	PV480507
2	*Boulenophrysdaxuemontis* sp.nov.	CIB WX20230630006	China, Yunan, Weixin County, Mt Daxue	PV469384	PV480508
3	*Boulenophrysdaxuemontis* sp.nov.	CIB WX20230630007	China, Yunan, Weixin County, Mt Daxue	PV469385	PV480509
4	*Boulenophrysdaxuemontis* sp.nov.	CIB JL20230630025	China, Sichuan, Junlian County, Mt Daxue	PV469386	PV480510
5	*acuta*	SYS a002266	China, Guangdong, Fengkai	KJ579119	MH406122
6	*angka*	KIZ 040591	Thailand, Chiang Mai, Doi Inthanon	MN508052	/
7	*anlongensis*	CIB AL20190531018	China, Guizhou, Anlong	MT823184	MT823261
8	*baishanzuensis*	CIB QY20200719002	China, Zhejiang, Qingyuan	MW001151	MT998292
9	*baolongensis*	KIZ 019216	China, Chongqing, Wushan	KX811813	KX812093
10	*binchuanensis*	KIZ 019441	China, Yunnan, Mt Jizu	KX811849	KX812112
11	*binlingensis*	KIZ 025807	China, Sichuan, Mt Wawu	KX811852	KX812115
12	*boettgeri*	KIZ YPXJK033	China, Fujian, Mt Wuyi	KX811814	KX812104
13	*brachykolos*	SYS a002258	China, Hong Kong	KJ560403	MH406120
14	*caobangensis*	IEBR 4385	Vietnam, Cao Bang, Nguyen Binh	LC483945	/
15	*caudoprocta*	SYS a004293	China, Hunan, Sangzhi	MH406796	MH406258
16	*cheni*	SYS a004050	China, Jiangxi, Mt Jinggang	MF667873	MH406241
17	*chishuiensis*	SYS a004953	China, Guizhou, Chishui	MH406867	MH406329
18	*congjiangensis*	GZNU 20200706003	China, Guizhou, Congjiang	MW959773	MW959761
19	*daiyunensis*	SYS a007711	China, Fujian, Tong’an	MW367054	MW365504
20	*daoji*	SYS a006212	China, Zhejiang, Mt Tiantai	MW367047	MW365497
21	*daweimontis*	KIZ 048997	China, Yunnan, Mt Dawei	KX811867	KX812125
22	*dongguanensis*	SYS a002007	China, Guangdong, Dongguan	MH406654	MH406090
23	*elongata*	GEP a150	China, Guangdong, Huizhou, Mt Lianhua	OR601592	OR597098
24	*fanjingmontis*	SYS a004350	China, Guizhou, Mt Fanjing	MH406808	MH406270
25	*fansipanensis*	AMS R186115	Vietnam, Lao Cai, Sapa	MH514887	MW086548
26	*fengshunensis*	SYS a004724	China, Guangdong, Fengshun	MH406848	MH406310
27	*frigida*	AMS R186131	Vietnam, Lao Cai, Bat Xat	MT364279	MW086550
28	*gaolanensis*	SYS a009225	China: Guangdong: Zhuhai: Gaolan Island	PQ217847	PQ218778
29	*hengshanensis*	CSUFT HS210612	China, Hunan, Mt Hengshan	ON209291	/
30	*hoanglienensis*	VNMN 2018.02	Vietnam, Lao Cai, Sapa	MH514889	MW086551
31	*hungtai*	SYS a007577	China, Guangdong, Jiexi	OL635594	OL634861
32	*insularis*	SYS a002171	China, Guangdong, Nan'ao	MH406665	MH406105
33	*jiangi*	CIB KKS20180722006	China, Guizhou, Kuankuoshui	MN107743	MN107748
34	*jiangi*	CIBKKS20180426001	China,Guizhou, Kuankuoshui	MN107744	MN107749
35	*jiangi*	CIBFJS20150720004	China,Guizhou,Fanjingshan	MN107747	MN107752
36	*jiangi*	CIBFJS20150719009	China,Guizhou,Fanjingshan	MN107746	MN107751
37	*jingdongensis*	SYS a003928	China, Yunnan, Mt Wuliang	MH406773	MH406232
38	*jinggangensis*	SYS a004028	China, Jiangxi, Mt Jinggang	MH406780	MH406239
39	*jiulianensis*	SYS a004218	China, Jiangxi, Mt Jiulian	MH406790	MH406252
40	*kuatunensis*	SYS a003449	China, Jiangxi, Mt Wuyi	MF667881	MH406206
41	*leishanensis*	KIZ 049172	China, Guizhou, Mt Leigong	KX811825	KX812102
42	*liboensis*	GNUG 20150813001	China, Guizhou, Libo	MF285253	/
43	*lichun*	CIB 121428	China, Fujian, Ningde	PQ309137	PQ300665
44	*lini*	KIZ 07053	China, Jiangxi, Mt Jinggang	KX811842	KX812110
45	*lishuiensis*	SYS a008445	China, Zhejiang, Liandu	OQ180984	OQ180872
46	*lushuiensis*	CIB YN201909289	China, Yunnan, Lushui	MW001226	MW000913
47	*minor*	KIZ YPX37545	China, Sichuan, Dujiangyan	KX811895	KX812144
48	*mirabilis*	SYS a002289	China, Guangxi, Lingui	MH406681	MH406127
49	*mufumontana*	SYS a006419	China, Hunan, Mt Mufu	MK524107	MK524138
50	*nankunensis*	SYS a004503	China, Guangdong, Mt Nankun	MH406824	MH406286
51	*nanlingensis*	SYS a001962	China, Guangdong, Ruyuan	MH406645	MH406081
52	*obesa*	SYS a002275	China, Guangdong, Fengkai	KJ579123	MH406125
53	*ombrophila*	CIB WY18082308	China, Fujian, Mt Wuyi	MW001159	MT998300
54	*omeimontis*	KIZ 025765	China, Sichuan, Mt Emei	KX811884	KX812136
55	*palpebralespinosa*	KIZ 011650	Vietnam, Thanh Hoa, Pu Hu	KX811889	KX812138
56	*pepe*	GEP a207	China, Guangdong, Qingyuan	PQ131151	PQ130479
57	*puningensis*	SYS a005770	China, Guangdong, Puning	OL635585	OL634853
58	*qianbeiensis*	CIB TZ20190608017	China, Guizhou, Tongzi	MT651554	MT654521
59	*rubrimera*	AMS R177676	Vietnam, Lao Cai, Sapa	MF536419	MW086542
60	*sangzhiensis*	SYS a004313	China, Hunan, Sangzhi	MH406802	MH406264
61	*sanmingensis*	SYS a007057	China, Jiangxi, Mt Magu	MW367051	MW365501
62	*shimentaina*	SYS a002077	China, Guangdong, Yingde	MH406655	MH406092
63	*shunhuangensis*	HNNU 16SH04	China, Hunan, Mt Shunhuang	MK836027	/
64	*spinata*	KIZ 016100	China, Guizhou, Mt Leigong	KX811864	KX812119
65	*tongboensis*	SYS a003227	China, Jiangxi, Mt Tongbo	MH406744	MH406201
66	*tuberogranulata*	KIZ YPX10987	China, Hunan, Sangzhi	KX811823	KX812095
67	*wugongensis*	SYS a004801	China, Jiangxi, Anfu	MH406854	MH406316
68	*wuliangshanensis*	SYS a003924	China, Yunnan, Mt Wuliang	MH406771	MH406230
69	*wushanensis*	KIZ YPX47799	China, Chongqing, Wushan	KX811835	/
70	*xiangnanensis*	SYS a002874	China, Hunan, Shuangpai	MH406713	MH406165
71	*xianjuensis*	CIB XJ190503	China, Zhejiang, Xianju	MN563758	MN563774
72	*xuefengmontis*	SYS a007227	China, Hunan, Mt Xuefeng	OQ180973	OQ180861
73	*yangmingensis*	SYS a002888	China, Hunan, Shuangpai	MH406719	MH406171
74	*yaoshanensis*	SYS a004878	China, Guangxi, Jinxiu	MH406863	MH406325
75	*yingdeensis*	SYS a004721	China, Guangdong, Yingde	MH406846	MH406308
76	*yunkaiensis*	SYS a004694	China, Guangdong, Xinyi	MH406845	MH406307
77	*Xenophrysmangshanensis*	SYS a002177	China, Guangdong, Huaiji	MH406666	MH406106
78	*Xenophrysglandulosa*	SYS a003757	China, Yunnan, Mt Gaoligong	MH406754	MH406213

**Table caption**: Specimens collected in this study are *permanently* deposited in the Chengdu Institute of Biology, Chinese Academy of Sciences(CIB，CAS).

**Table 2.** References for morphological characters for congeners of the genus *Boulenophrys*.

**ID**	**Species**	**References**
1	*B.acuta* Wang, Li & Jin, 2014	Li et al. 2014
2	*B.angka* Wu, Suwannapoom, Poyarkov, Pawangkhanant, Xu, Jin, Murphy & Che, 2019	Wu et al. 2019
3	*B.anlongensis*Li, Lu, Liu & Wang, 2020	Li et al. 2020
4	*B.baishanzuensis* Wu, Li, Liu, Wang & Wu, 2020	Wu et al. 2020
5	*B.baolongensis* Ye, Fei & Xie, 2007	Ye et al. 2007
6	*B.binchuanensis* Ye & Fei, 1995	Ye and Fei 1995
7	*B.binlingensis* Jiang, Fei & Ye, 2009	Fei et al. 2009
8	*B.boettgeri* Boulenger, 1899	Boulenger 1899
9	*B.brachykolos* Inger & Romer, 1961	Inger and Romer 1961
10	*B.caobangensis* Nguyen, Pham, Nguyen, Luong & Ziegler, 2020	Nguyen et al. 2020
11	*B.caudoprocta* Shen, 1994	Shen 1994
12	*B.cheni* Wang & Liu, 2014	Wang et al. 2014
13	*B.chishuiensis* Xu, Li, Liu, Wei & Wang, 2020	Xu et al. 2020
14	*B.congjiangensis* Luo, Wang, Wang, Lu, Wang, Deng & Zhou, 2021	Luo et al. 2021
15	*B.daiyunensis* Lyu, Wang & Wang, 2021	Lyu et al. 2021
16	*B.daoji* Lyu, Zeng, Wang & Wang, 2021	Lyu et al. 2021
17	*B.daweimontis* Rao & Yang, 1997	Rao and Yang 1997
18	*B.dongguanensis* Wang & Wang, 2019	Wang et al. 2019
19	*B.dupanglingensis* Xiao & Mo, 2025	Xiao et al. 2025
20	*B.elongata* Zeng, Wang, Chen, Xiao, Zhan, Li & Lin, 2024	Zeng et al. 2024
21	*B.fanjingmontis* Zhang, Liang, Ran & Shen, 2012	Zhang et al. 2012
22	*B.fansipanensis* Tapley, Cutajar, Mahony, Nguyen, Dau, Luong, Le, Nguyen,Nguyen, Portway, Luong & Rowley, 2018	Tapley et al. 2018
23	*B.fengshunensi* Wang, Zeng, Lyu & Wang, 2022	Wang et al. 2022
24	*B.frigida* Tapley, Cutaja, Nguyen, Portway, Mahony, Nguyen, Harding, Luong& Rowley, 2021	Tapley et al. 2021
25	*B.gaolanensis* Song, Wang,Qi, Wang, Wang,2024	Song et al. 2024
26	*B.hengshanensis* Qian, Hu, Mo, Gao, Zhang & Yang, 2023	Qian et al. 2023
27	*B.hoanglienensis* Tapley, Cutajar, Mahony, Nguyen, Dau, Luong, Le, Nguyen,Nguyen, Portway, Luong & Rowley, 2018	Tapley et al. 2018
28	*B.hungtai* Wang, Zeng, Lyu, Xiao & Wang, 2022	Wang et al. 2022
29	*B.insularis* Wang, Liu, Lyu, Zeng & Wang, 2017	Wang et al. 2017
30	*B.jiangi* Liu, Li, Wei, Xu, Cheng, Wang & Wu, 2020	Liu et al. 2020
31	*B.jingdongensis* Fei & Ye, 1983	Fei et al. 1983
32	*B.jinggangensis* Wang, 2012	Wang et al. 2012
33	*B.jiulianensis* Wang, Zeng, Lyu & Wang, 2019	Wang et al. 2019
34	*B.kuatunensis* Pope, 1929	Pope 1929
35	*B.leishanensis* Li, Xu, Liu, Jiang, Wei & Wang, 2018	Li et al. 2018
36	*B.liboensis* Zhang, Li, Xiao, Li, Pan, Wang, Zhang & Zhou, 2017	Zhang et al. 2017
37	*B.lichun* Lin, Chen, Li, Peng, Zeng, Wang, 2024	Lin et al. 2024
38	*B.lini* Wang & Yang, 2014	Wang et al. 2014
39	*B.lishuiensis* Wang, Liu & Jiang, 2017	Wang et al. 2017
40	*B.lushuiensis* Shi, Li, Zhu, Jiang, Jiang & Wang, 2021	Shi et al. 2021
41	*B.minor* Stejneger, 1926	Stejneger 1926
42	*B.mirabilis* Lyu, Wang & Zhao, 2020	Lyu et al. 2020
43	*B.mufumontana* Wang, Lyu & Wang, 2019	Wang et al. 2019
44	*B.nankunensis* Wang, Zeng & Wang, 2019	Wang et al. 2019
45	*B.nanlingensis* Lyu, Wang, Liu & Wang, 2019	Lyu et al. 2019
46	*B.obesa* Wang, Li & Zhao, 2014	Li et al. 2014
47	*B.ombrophila* Messenger & Dahn, 2019	Messenger et al. 2019
48	*B.omeimontis* Liu, 1950	Liu 1950
49	*B.palpebralespinosa* Bourret, 1937	Bourret 1937
50	*B.pepe* Wang & Zeng, 2024	Wang et al. 2024
51	*B.puningensis* Wang, Zeng, Lyu, Xiao & Wang, 2022	Wang et al. 2022
52	*B.qianbeinsis* Su, Shi, Wu, Li, Yao, Wang & Li, 2020	Su et al. 2020
53	*B.rubrimera* Tapley, Cutajar, Mahony, Chung, Dau, Nguyen, Luong & Rowley,2017	Tapley et al. 2017
54	*B.sangzhiensis* Jiang, Ye & Fei, 2008	Jiang et al. 2008
55	*B.sanmingensis* Lyu & Wang, 2021	Lyu et al. 2021
56	*B.shimentaina* Lyu, Liu & Wang, 2020	Lyu et al. 2020
57	*B.shuichengensis* Tian & Sun, 1995	Tian and Sun 1995
58	*B.shunhuangensis* Wang, Deng, Liu, Wu & Liu, 2019	Wang et al. 2019
59	*B.spinata* Liu & Hu, 1973	Hu et al. 1973
60	*B.tongboensis* Wang & Lyu, 2021	Lyu et al. 2021
61	*B.tuberogranulatus* Shen, Mo & Li, 2010	Mo et al. 2010
62	*B.wugongensis* Wang, Lyu & Wang, 2019	Wang et al. 2019
63	*B.wuliangshanensis* Ye & Fei, 1995	Ye and Fei 1995
64	*B.wushanensis* Ye & Fei, 1995	Ye and Fei 1995
65	*B.xiangnanensis* Lyu, Zeng & Wang, 2020	Lyu et al. 2020
66	*B.xianjuensis* Wang, Wu, Peng, Shi, Lu & Wu, 2020	Wang et al. 2020
67	*B.xuefengmontis* Lyu & Wang, 2023	Lyu et al. 2023
68	*B.yangmingensis* Lyu, Zeng & Wang, 2020	Lyu et al. 2020
69	*B.yaoshanensis* Qi, Mo, Lyu, Wang & Wang, 2021	Qi et al. 2021
70	*B.yingdeensis* Qi, Lyu, Wang & Wang, 2021	Qi et al. 2021
71	*B.yunkaiensis* Qi, Wang, Lyu & Wang, 2021	Qi et al. 2021

**Table 3.** Measurements of the adult specimens of *Boulenophrysdaxuemontis* sp. nov. and *B.jiangi*. Units are in mm. See abbreviations for the morphological characters in Materials and Methods section.

ID	Species	Voucher number	Sex	SVL	HDL	HDW	SL	IND	IOD	UEW	ED	TYD	LAL	LW	HAL	HLL	THL	TL	TW	TFL	FL
1	*B.daxuemontis* sp.nov.	CIB WX20230630006	Male	37.58	10.67	12.04	4.46	4.27	3.51	3.36	3.54	2.77	16.49	2.43	9.40	53.99	15.49	17.02	3.56	25.16	14.83
2	*B.daxuemontis* sp.nov.	CIB JL20230630024	Male	37.69	10.72	11.67	4.20	4.58	3.52	3.58	4.15	2.34	15.88	2.30	8.59	54.41	15.52	16.67	3.46	24.35	15.13
3	*B.daxuemontis* sp.nov.	CIB WX20230630026	Male	40.55	11.05	12.35	5.26	4.50	3.89	3.99	3.91	3.04	17.38	3.02	10.07	60.79	15.85	18.92	4.27	27.23	15.82
4	*B.daxuemontis* sp.nov.	CIB WX20230630007	Male	37.69	11.03	11.33	4.80	4.61	3.87	3.09	4.17	2.88	16.20	2.88	8.94	54.50	16.67	17.42	3.52	24.28	14.50
5	*B.daxuemontis* sp.nov.	CIB JL20230630025	Male	37.06	10.84	12.41	4.87	4.61	4.15	3.19	4.21	2.54	16.88	3.26	9.19	57.87	15.86	16.16	3.33	25.56	16.58
6	*B.jiangi*	kks20210605007	Male	36.96	9.88	12.21	4.16	3.54	2.96	3.65	4.73	2.23	16.49	2.85	9.07	53.11	16.65	18.32	3.58	23.71	16.41
7	*B.jiangi*	kks20180720001	Male	37.30	10.51	12.06	4.12	4.23	3.10	3.82	4.52	2.56	13.83	2.07	8.44	53.73	16.24	17.16	3.88	22.80	16.27
8	*B.jiangi*	kks20180723002	Male	38.37	11.86	12.95	4.86	4.29	3.11	4.67	5.33	2.44	18.69	2.94	9.03	56.70	17.88	18.93	3.89	24.77	17.29
9	*B.jiangi*	kks20180723004	Male	38.66	11.54	11.94	4.39	4.07	2.91	4.15	5.13	2.79	15.77	3.09	8.95	60.05	18.65	19.17	4.31	27.22	16.23
10	*B.jiangi*	kks20180723007	Male	37.60	10.40	12.21	4.05	4.24	3.28	3.72	5.51	3.09	14.89	2.74	8.65	51.08	17.92	17.91	3.88	21.90	15.63
11	*B.jiangi*	kks20180723008	Male	34.45	10.01	10.39	4.16	4.05	3.27	3.71	4.47	2.36	14.34	2.78	9.17	51.08	15.87	16.97	3.48	20.83	15.19
12	*B.jiangi*	kks20180723010	Male	39.33	11.60	11.93	4.83	4.44	3.25	3.65	5.23	2.59	17.14	2.56	8.90	58.67	18.09	18.98	4.97	23.25	16.09
13	*B.jiangi*	kks20180723011	Male	34.82	10.00	11.44	4.08	4.15	2.83	3.67	5.04	2.32	15.73	3.10	9.07	51.83	16.09	17.79	3.94	22.78	15.24

**Table 4.** Morphometric comparisons between *Boulenophrysdaxuemontis* sp. nov. and *B.jiangi*.

Character	*Boulenophrysdaxuemontis* sp. nov.		*B.jiangi*		p-value from ANOVA in male
	Male (n=5)		Male (n=8)		
	Range	Mean ± SD	Range	Mean ± SD	
SVL	37.06-40.55	38.11±1.39	34.45-39.33	37.19±1.75	0.186
HDL	10.67-11.05	10.86±0.17	9.88-11.86	10.73±0.81	0.092
HDW	11.33-12.41	11.96±0.46	10.39-12.95	11.89±0.74	0.265
SL	4.20-5.26	4.72±0.41	4.05-4.86	4.33±0.33	0.193
IND	4.27-4.61	4.514±0.14	3.54-4.44	4.13±0.27	0.382
IOD	3.51-4.15	3.79±0.27	2.83-3.28	3.09±0.17	0.01
UEW	3.09-3.99	3.44±0.36	3.65-4.67	3.88±0.36	0.159
ED	3.54-4.21	4.00±0.28	4.47-5.51	5.00±0.38	0.001
TYD	2.34-3.04	2.71±0.28	2.23-3.09	2.55±0.28	0.825
LAL	15.88-17.38	16.57±0.59	13.83-18.69	15.86±1.58	0.534
LW	2.30-3.26	2.78±0.40	2.07-3.1	2.77±0.33	0.969
HAL	8.59-10.07	9.24±0.55	8.44-9.17	8.91±0.25	0.982
HLL	53.99-60.79	56.31±2.95	51.08-60.05	54.53±3.51	0.497
THL	15.49-16.67	15.88±0.48	15.87-18.65	17.17±1.08	0.074
TL	16.16-18.92	17.24±1.05	16.97-19.17	18.15±0.84	0.031
TW	3.33-4.27	3.63±0.37	3.48-4.97	3.99±0.47	0.194
TFL	24.28-27.23	25.32±1.20	20.83-27.22	23.41±1.93	0.839
FL	14.50-16.58	15.37±0.83	15.19-17.29	16.04±0.69	0.019

**Table 5.** Table 5. Factor loadings of the first two principal components for 18 size-adjusted male morphometric characteristics of *Boulenophrysdaxuemontis* sp.nov. and *B.jiangi*

Character	PC1	PC2
Eigenvalue	5.186	3.822
% variation	28.814	21.233
SVL	-0.339	-0.285
HDL	0.246	0.439
HDW	0.356	0.309
SL	-0.38	0.708
NED	-0.47	0.321
NSD	-0.794	0.408
IND	0.774	0.081
IOD	0.902	-0.06
ED	-0.336	-0.291
UEW	0.12	0.741
LAL	0.283	0.768
LW	0.03	0.617
HLL	0.004	0.698
THL	0.834	-0.05
TL	0.871	0.051
TW	0.527	-0.252
TFL	-0.271	0.582
FL	0.682	0.415
